# Understanding heterogeneity of investor sentiment on social media: A structural topic modeling approach

**DOI:** 10.3389/frai.2022.884699

**Published:** 2022-10-06

**Authors:** Rongjiao Ji, Qiwei Han

**Affiliations:** ^1^Department of Mathematics, University of Milan, Milan, Italy; ^2^Nova School of Business and Economics, Universidade NOVA de Lisboa, Lisbon, Portugal

**Keywords:** investor sentiment, structural topic modeling, text mining, social media, unstructured data analysis

## Abstract

Investors nowadays post heterogeneous sentiments on social media about financial assets based on their trading preferences. However, existing works typically analyze the sentiment by its content only and do not account for investor profiles and trading preferences in different types of assets. This paper explicitly considers how investor sentiment about financial market events is shaped by the relative discussions of different types of investors. We leverage a large-scale financial social media dataset and employ a structural topic modeling approach to extract topical contents of investor sentiment across multiple finance-specific factors. The identified topics reveal important events related to the financial market and show strong heterogeneity in the social media content in terms of compositions of investor profiles, asset categories, and bullish/bearish sentiment. Results show that investors with different profiles and trading preferences tend to discuss financial markets with heterogeneous beliefs, leading to divergent opinions about those events regarding the topic prevalence and proportion. Moreover, our findings may shed light on the mechanism that underlies the efficient investor sentiment extraction and aggregation while considering the heterogeneity of investor sentiment across different dimensions.

## 1. Introduction

Investors' noise trading decisions are known to be subject to their sentiment or perception about the financial market, which in turn leads to more noise trading behavior, and excess financial market volatility (DeLong et al., 1990). The assessment and measurement of investor sentiment thus have become an important research topic to evaluate its effects on the financial market (Baker and Wurgler, 2007). As such, researchers increasingly leverage a variety of financial data sources and employ computational approaches to extract investor sentiment from the unstructured textual information, such as financial news media (Tetlock et al., 2008; Boudoukh et al., 2013; Heston and Sinha, 2017), Internet stock message board (Tumarkin and Whitelaw, 2001; Hou and Tripathi, 2015), and social media (Xu and Zhang, 2013; Wang et al., 2015).

In particular, social media recently continues to expand the user base and reach more audiences in the finance industry. In April 2013, the U.S. Securities and Exchange Commission (SEC) confirmed that companies could use social media outlets like Twitter to announce key information in compliance with Regulation Fair Disclosure (U.S. Securities and Exchange Commission, 2013). Financial data vendors such as Bloomberg and Thompson Reuters also introduced social media monitoring and analytics tools to their professional workstations, which allow financial professionals and asset managers to capture alpha from growing financial discussions on social media platforms through sentiment analysis. Gan et al. (2020) find that although news media is traditionally the dominant source of investor sentiment, social media tends to generate and lead news coverage since 2016 (Ren et al., 2021). One notable example emerges as the recent heated discussion of “meme stocks” such as $GME (GameStop) and $AMC (AMC Entertainment) on social media platform Reddit, that investor sentiment was found to profoundly affect stock prices as well as news coverage (Long et al., 2021). Compared to sentiment extracted from news media, numerous studies show that investor sentiment on social media is better in predicting contemporaneous stock market returns (Deng et al., 2018; Lachana and Schröder, 2022).

Typically, investor sentiment on social media is extracted from user posts and aggregated at the asset level (Das and Chen, 2007), and it has been widely used to demonstrate its predictability of financial market performance, such as the trends of Dow Jones or S&P 500 Index (Bollen et al., 2011; Zheludev et al., 2014; Piñeiro-Chousa et al., 2016), stock price movement (Oh and Sheng, 2011; Zhang et al., 2012; Wang et al., 2015), abnormal returns (Ranco et al., 2015; Deng et al., 2018), earning surprises (Chen et al., 2014; Bartov et al., 2018), trading volume (Tan and Tas, 2021), and market volatility (Hou and Tripathi, 2015; Audrino et al., 2020). However, mixed evidence is also reported that investor sentiment does not have strong predictability of stock returns (Oliveira et al., 2013; Kim and Kim, 2014), or the magnitude of the effect is economically small (Nofer and Hinz, 2014).

Moreover, investor sentiment may have significant heterogeneous effects on the financial market that is not accounted for by current practices of sentiment aggregation. For example, stocks of low capitalization, younger, high-volatility, and growth companies are more likely to be disproportionately sensitive to investor sentiment (Baker and Wurgler, 2007; Dandapani et al., 2008; Zheludev et al., 2014; Bartov et al., 2018). Meanwhile, negative investor sentiment tends to produce a stronger influence on stock returns than positive sentiment (Heston and Sinha, 2017; Deng et al., 2018). Similarly, the heterogeneity of investor sentiment can also be observed according to investors' profiles and trading preferences. For example, given that social media may improve the information environment for investors in the financial market (Xu and Zhang, 2013), and investment strategies based on crowd sentiment from social media may outperform professional analysts (Nofer and Hinz, 2014), trading decisions made by sentiment aggregations extracted from “expert” investors with more trading experience may further outperform the entire crowd on social media (Bar-Haim et al., 2011; Hill and Ready-Campbell, 2011). This is due to the fact that social media platform contains unreasonable or misleading information posted by non-experts (Tu et al., 2016). Also, several studies find that investors who are more informed about local companies than non-local companies are more likely to post value-relevant private information on social media that influences the trading activity of local stocks (Baik et al., 2016; Giannini et al., 2018). Last, sentiment from investors with different investment philosophies, like investment approach and horizon, is an important source of disagreement, which in turn affects financial markets (Cookson and Niessner, 2020). Therefore, accounting for investors' profiles and trading preferences allows financial professionals to analyze their heterogeneous sentiment, leading to an efficient aggregation of information on social media (Sprenger et al., 2014).

In this work, we aim to understand the heterogeneity of online discussions about investment-related events on social media from different types of investors. We do so by exploring the interactions between investor sentiment and a set of finance-specific factors. Analyzing a unique large-scale dataset containing millions of tweets posted by investors enriched with their profiles, such as trading experience, trading strategy as well as categories of the mentioned assets, we employ a modified topic modeling approach, known as *structural topic modeling* (STM) to identify thematic information of investor sentiment with variations in topic prevalence (Roberts et al., 2016). The identified topics reveal important events related to the financial market with strong heterogeneity in terms of compositions of investor profiles, asset categories, and bullish/bearish sentiment, which are reflected by the large divergence on the topics they discuss.

We have two main contributions to the existing literature about extracting investor sentiment from increasingly available web-based sources. First, we incorporate the financial domain knowledge into the content analysis of investor sentiment and demonstrate that online discussions about investment-related events may be shaped by profiles and trading preferences of different types of investors with heterogeneous beliefs about those events. Second, we explore investor sentiment from user-generated social media content on a wide range of financial assets, including stocks, foreign exchange, and investment funds, and how they are co-mentioned in different events in terms of heterogeneous topic coverage and proportions. Overall, our results may shed light on the mechanism that underlies the efficient investor sentiment extraction and aggregation while taking into account the heterogeneity of investors' beliefs.

The rest of the paper is organized as follows. Section 2 reviews literature that studies investor sentiment with textual analysis and applications topic modeling in finance research. Section 3 introduces the dataset used in this research and performs explorative data analysis. Section 4 details the methodology in three steps: data preprocessing, aggregation, and structural topic modeling. Section 5 shows empirical results, including topical content as well as heterogeneous investor sentiment across several dimensions. Section 6 discusses research implications and concludes.

## 2. Related work

### 2.1. Investor sentiment with textual analysis

Overall, Loughran and Mcdonald (2016) provided comprehensive reviews on how to measure investor sentiment using textual analysis in the accounting and finance community and listed two main approaches: the dictionary-based and classifier-based methods. The former approach indicates that positive and negative words of documents are extracted from a dictionary. Such dictionary may be a generic dictionary or is refined to entail more finance-contextual definitions (Tetlock, 2007; Loughran and McDonald, 2011; Li et al., 2014). For example, Tetlock et al. (2008) measured investor sentiment by counting the fraction of negative words in news articles. Similarly, Bartov et al. (2018) constructed aggregate investor sentiment from tweets using several different dictionaries and provided consistent supporting evidence that investor sentiment may help to predict earning surprises.

The latter approach implements classifiers to assign documents with positive/negative or bullish/bearish labels through supervised machine learning models. This requires enough documents to be pre-labeled in order to build a good training set. For example, most studies started by manually labeling documents as bullish, bearish, or neutral and trained various machine learning models to predict the sentiment for other documents (Antweiler et al., 2004; Deng et al., 2018). Although dictionary-based methods were not obliged to human-defined ground truth, classifier-based methods were increasingly adopted to automatically predict investor sentiment from large-scale text data, such as Naïve Bayes (Bartov et al., 2018), Decision Tree (Nasseri et al., 2015), Maximum Entropy classifier (Giannini et al., 2019), Support Vector Machine (Ranco et al., 2015), and more recently developed Deep Neural Network models (Heston and Sinha, 2017; Audrino et al., 2020). Still, investor investment extracted from textual data using both dictionary-based and model-based methods may contain inherent errors. Instead, this study used the self-labeled sentiment from the investors directly.

### 2.2. Topic modeling in financial studies

Topic modeling such as Latent Dirichlet Allocation (LDA) is an unsupervised probabilistic model for extracting the latent thematic content within a collection of documents (Blei et al., 2003). It has also been increasingly adopted in the finance domain, such as identifying co-movement of stocks from stock price changes (Doyle and Elkan, 2009), characterizing abnormal financial market volatility from business news (Hisano et al., 2013; Curme et al., 2017), generating domain expert profiles (Siehndel and Gadiraju, 2016), discovering information from analyst reports and corporate disclosures (Huang et al., 2018), detecting regulatory evolution and financial misreporting from 10-K filings (Dyer et al., 2017; Brown et al., 2020), among others.

Several extensions of LDA models that alleviate the independence assumption among topics have also been applied to financial text data. For example, topics and investor sentiment can be inferred simultaneously from social media information using correlated topic models to predict stock movement (Nguyen and Shirai, 2015). Also, Mai and Pukthuanthong (2021) adopt a semi-supervised LDA approach to extract economic narratives from New York Times articles, by providing seed words for each topic to guide the formation of topics toward the predefined themes. Using the STM approach, Cerchiello and Nicola (2018) evaluated the news contagion across space and time dimensions with explicit inclusion of covariates such as involved country and publication date.

### 2.3. Topic modeling with metadata

In order to correlate the topics extracted from textual data to the features of metadata at the document level, many derivatives of LDA have been proposed, such as the supervised versions of LDA (Mcauliffe and Blei, 2007; Ramage et al., 2009; Zhu et al., 2012), dynamic topic models (Blei and Lafferty, 2006; Wang et al., 2012). Alternatively, Multinomial Inverse Regression (MNIR) focuses on the influence of document-level response variable on the multinomial distribution of words and the word selection process (Taddy, 2013). MNIR may use the sentiment as the observed response variable that may shape the composition of the text data. It is effective in capturing the predictive information about the response but fails to capture the underlying semantic themes within the corpus (Li et al., 2017). In comparison to MNIR, Roberts et al. (2020) show that STM has the form of MNIR conditional on the latent variable and both approaches may yield qualitatively similar findings (Besbris et al., 2021).

As a mixed-membership extension of MNIR and LDA, the Inverse Regression Topic Model (IRTM) combines the strengths of topic modeling with the expressive power by accounting for the heterogeneity of text corpora and the context related to topic expression (Rabinovich and Blei, 2014). IRTM enables systematic discovery of in-topic lexical variation and exploits the topical structure of text corpora to improve its predictions. However, the inference procedure is rather complex and inefficient (Rodrigues et al., 2017).

## 3. Data

**Figure 1:**
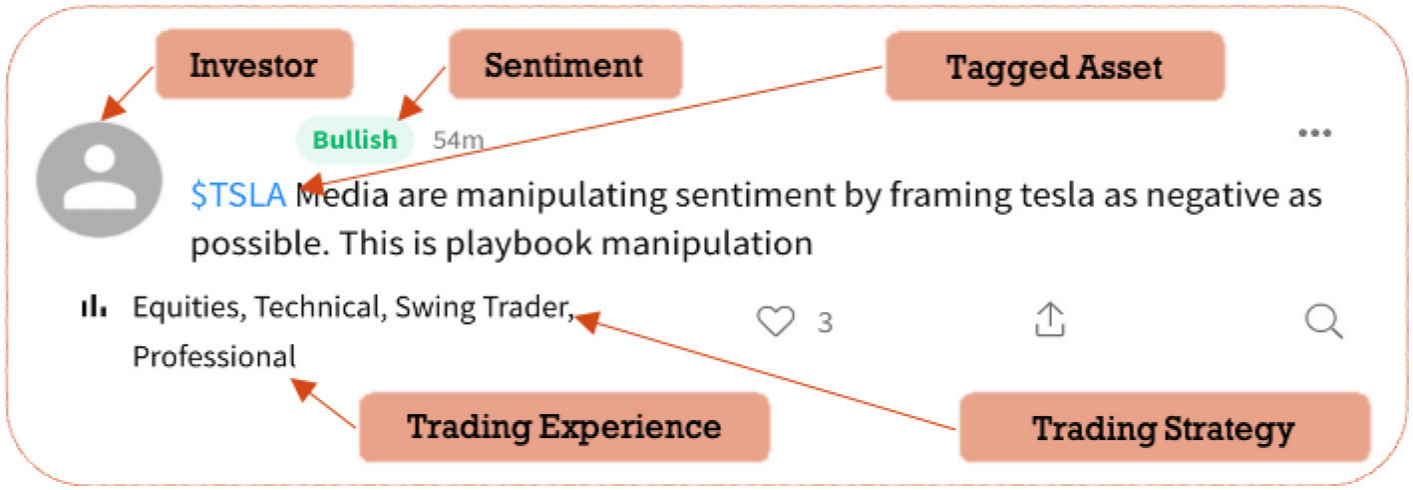
An illustrative tweet posted by an investor with a detailed trading profile such as trading experience and trading strategy on StockTwits platform, clarifying also the tagged asset under discussion and the self-labeled investor sentiment.

The dataset used for this research includes 5,286,637 user posts from StockTwits spanning from March 2010 to August 2015. StockTwits is a financial social media platform dedicated to financial assets-related discussions, and also a social network between investors to exchange their opinions (Oh and Sheng, 2011). As of today, StockTwits has 3 million registered members, with 5 million messages sent monthly through the platform. Similar to Twitter but specifically for investors and traders to discuss the financial market, a typical user post, like a tweet, is limited to 140 characters. Figure 1 shows an illustrative tweet on the StockTwits platform. Financial discussions on StockTwits are enabled through the convention of cashtag. Users can associate a discussion with tradable assets using a cashtag followed by its ticker symbol (“$TICKER”). Cashtag allows StockTwits users to easily identify sentiment for a specific stock. Unlike other social media platforms, where a search for “Apple” yields scattered and often irrelevant results, searching for “$AAPL” on StockTwits pulls up conversations only regarding financial information about Apple Inc. (Oliveira et al., 2013). Also, self-reported investor profiles are available for all users, including their trading experience (novice, intermediate, professional), and trading strategy (day trader, swing trader, position trader, long-term investor). Meanwhile, when posting a tweet about one or more assets, the user is allowed to assign bullish/bearish sentiment herself, which can be considered as the ground-truth sentiment (Fallahgoul and Lin, 2020). We believe that self-labeled bullish/bearish sentiments are better measurements of investor sentiment than those derived from the textual analysis that may contain errors and does not reflect investors' own opinions. Last, we obtain categories of all ticker symbols that cover a wide range of assets directly from StockTwits, such as Foreign Exchange (Forex), Stock Index, Exchange-traded funds (ETF), Closed-end funds (CEF), as well as eight industry sectors of stocks^1^. Overall, the dataset used in this work is enriched with a variety of metadata describing both investors and assets, which allows us to characterize investor sentiment subject to heterogeneous investor profiles and trading preferences.

As demonstrated in extant previous studies, value-relevant signals have been extracted from financial discussions on StockTwits to predict future stock movements (Bollen et al., 2011; Oh and Sheng, 2011; Nasseri et al., 2015), volatility and trading volume (Oliveira et al., 2013) and stock returns (Deng et al., 2018), as well as to investigate disagreement and behavior contagions among investors (Li et al., 2016; Cookson and Niessner, 2020; Fallahgoul and Lin, 2020). Typically, investor sentiment is measured at aggregated level to study the relationship between sentiment index and asset performance (Piñeiro-Chousa et al., 2016; Tu et al., 2016), while this work focuses on exploring the differences in investor sentiment across different types of investors and assets.

**Figure 2:**
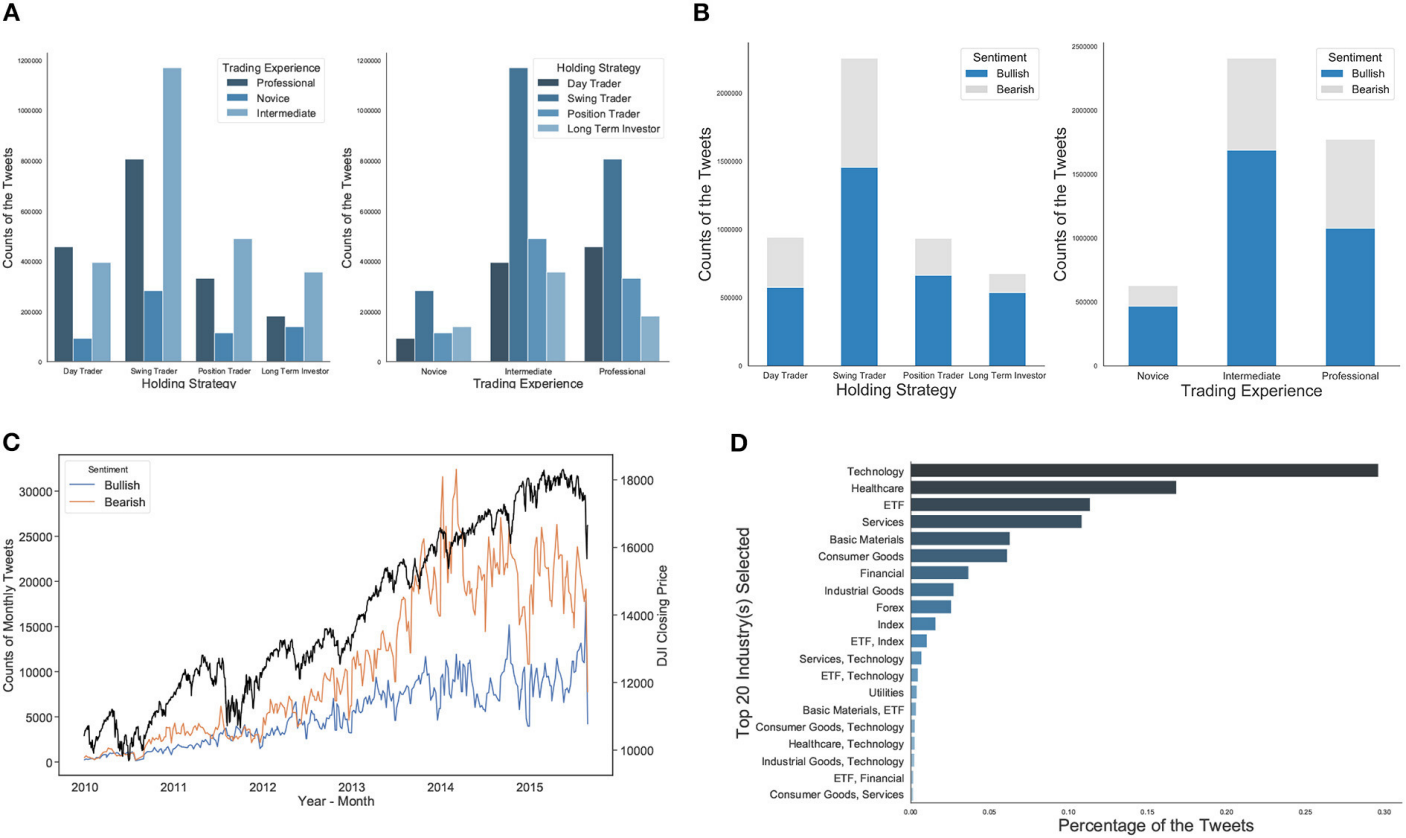
**(A)** The distribution of posted tweets under the consideration of investors' trading experience and holding strategy. Swing traders who aim to capture short- to medium-term gains over a few days and weeks contribute the most number of tweets. **(B)** The distribution of self-labeled bullish/bearish tweets on different factors of investors' trading experience and strategy. There are more bullish tweets than bearish ones. Meanwhile, investors with intermediate experience (indicating a certain level of financial literacy) post the most number of tweets. **(C)** The trends of the monthly amounts of bullish/bearish tweets in comparison to Dow Jones Index (DJI) closing prices over the same period, demonstrating the differences between bullish and bearish investor sentiment follow similar trends of the DJI movement. **(D)** Top 20 asset categories discussed the most, listed by the number of associated tweets. Technology stocks, healthcare stocks, ETFs are among the top 3 most mentioned asset categories on StockTwits.

We start with exploring the dataset across several dimensions with respect to the heterogeneity of investor sentiment. Figure 2A shows that swing traders who aim to capture short- to medium-term gains over a few days and weeks contribute the most number of tweets, followed by day traders, position traders, and long-term traders. Likewise, Figure 2B shows that investors with intermediate experience post the most number of tweets, followed by professional investors and novice investors. This indicates that StockTwits may have a significant number of investors with a certain level of financial literacy. We also observe that novice investors post much more bullish tweets than bearish ones, while intermediate investors and professional investors post a more balanced sentiment. This implies that investor sentiment may have a positivity bias, conditional on trading experience.

As numerous studies using StockTwits dataset demonstrated that it may contain value-relevant information for financial market performance (Oh and Sheng, 2011; Deng et al., 2018; Cookson and Niessner, 2020), Figure 2C shows the number of bullish/bearish tweets in comparison to Dow Jones Index (DJI) closing prices over the same period. We can see that the differences between bullish and bearish investor sentiment follow similar trends in the DJI movement. Figure 2D lists the number of tweets by the top 20 asset categories. About 93% of tweets contain assets from one asset category and 4.7% of tweets contain assets from two asset categories. For example, technology stocks, healthcare stocks, and ETFs are among the top 3 most mentioned asset categories on StockTwits, and this is consistent with findings from existing literature that investor attention and discussions tend to distribute disproportionately toward a small set of assets, such as AAPL (Apple), FB (Facebook), TSLA (Tesla), SPY (S&P 500 ETF), etc. (Cookson and Niessner, 2020).

## 4. Methods

### 4.1. Preprocessing and aggregation

To ensure that we can identify meaningful topics from tweets posted on StockTwits, we perform several preprocessing steps to extract the subsets of the original dataset and augment and aggregate tweets according to the metadata detailed below.

#### 4.1.1. Enrich the tweets with metadata

For all tweets in the dataset, we augment them with a variety of metadata. First, tweets are linked with investor profiles such as trading experience, holding strategy as well as the self-labeled sentiment. Moreover, since we are only interested in studying investor sentiment for assets, the social tweets between users that do not mention any asset are deemed irrelevant (e.g., retweet and reply). Therefore, we only retain tweets that contain at least one ticker symbol. The ticker symbol is also cross-referenced with the asset categories that StockTwits has officially maintained. In particular, for tweets with more than one ticker, we generate the category combination if these tickers are from different categories. For example, if one tweet mentions both AAPL and SPY, it will be associated with the category of [Technology, ETF]. We only keep tweets that belong to the top 20 asset categories. Last, we further add the temporal dimension to the metadata of tweets using its timestamp.

#### 4.1.2. Text preprocessing

Standard text preprocessing techniques have been applied to all tweets, such as removing all numbers, non-ASCII notations, stop words and links, converting all characters to be lowercase, and eliminating tweets whose word length is less than two, and using the stemming and lemmatization method to obtain the root form of the words. Then, terms with a relatively high sparsity level of 0.995 are removed in order to keep only meaningful and frequent words in tweets.

#### 4.1.3. Remove robots

From the exploratory data analysis, we identify many news bots (hereby referred to as “robots”) that tweet about a broad coverage of assets using technical terminologies similarly. We choose to remove these robots from the data because they do not represent actual investor sentiment and may distort the results. More specifically, we first select the user IDs that have posted the top 1000 highest number of tweets. Then we compute the average similarity score of 100 pairs of randomly chosen tweets for each ID using fuzzy string matching. We consider users a robot if the average similarity is above a threshold (set as 0.6), thus removing their tweets from our corpus. As such, 4,590,854 tweets remain after we finish preprocessing steps.

#### 4.1.4. Aggregate the tweets by metadata

Topic modeling is known to produce less coherent topics when directly applying to short texts such as tweets due to the lack of word co-occurrence information in each short text (Hong and Davison, 2010). We follow a widely adopted pooling scheme that utilizes auxiliary contextual information to aggregate related tweets and organizes them as a single regular-sized document before performing topic modeling (Mehrotra et al., 2013). In particular, we aggregate all tweets into 27,189 documents according to metadata built earlier, including trading experience, holding strategy, investor sentiment, asset category at the monthly level.

### 4.2. Structural topic modeling

Compared with the classical topic model, STM extends the clustering ability and explanatory power by further incorporating the core language model with the document-level metadata, rather than only relying on words in the documents (Roberts et al., 2016). In recent years, several empirical studies applying STM on social media data have emerged in various social science areas, e.g., to perform a descriptive analysis of Twitter conversations about the reasons individuals choose to leave or stay in abusive relationship (Rodriguez and Storer, 2020), to explore the content and dynamics of media coverage of Internet regulation in Russia (Shirokanova and Silyutina, 2018) and to understand how elected state high court judges use social media to engage with the public (Curry and Fix, 2019), among others.

Although LDA assumes that the topic distributions of the documents, viewed as topic prevalence, have the common Dirichlet priors internally (Blei et al., 2003), the distributions of those parameters associated with the sources and the idioms of the documents may vary largely in practice. Instead, STM considers that the attributes of document-level metadata can influence the modeling process in terms of topic prevalence and topical content. Hence the original assumption of one common set of parameters distributions may not uncover heterogeneous characteristics of document-level covariates. The topic prevalence of STM is a function of covariates characterizing documents, giving the model explanatory power to express the relationship between document characteristics and topic prevalence (Roberts et al., 2016). Likewise, the topical content of STM takes into account covariates similarly, which is parameterized as the distribution of word occurrences devoted to a topic from the proportion of terms in the general corpus. In our setting, we consider the covariates of the investor sentiment aggregated at investors' trading experience, holding strategy, asset category, bullish/bearish sentiment, and posted timestamp. In particular, investors' trading experience is used as the covariate in topical content by assuming that investors with different levels of experience may use diverse terminologies, while other covariates are used in topic prevalence. For example, novice investors tend to use emotional and non-technical words, while professional investors prefer more technical and macro terms.

#### 4.2.1. The data generative process

**Figure 3:**
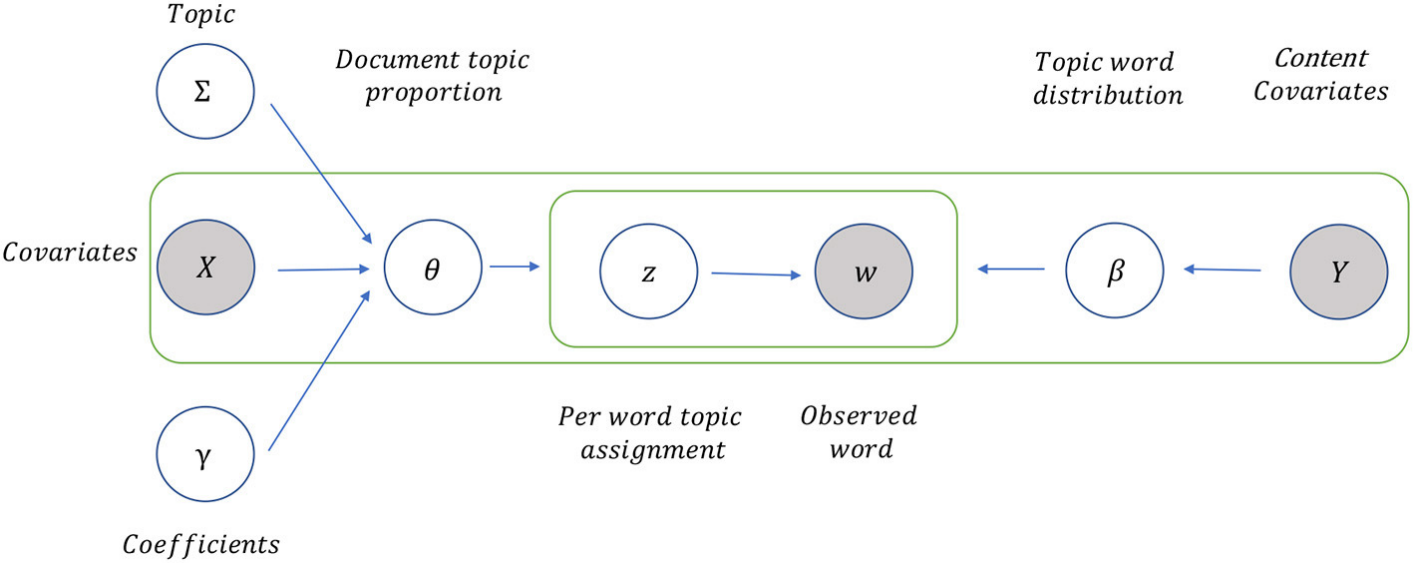
A graphical illustration of data generative process of structural topic modeling (STM). It assumes that the attributes of document-level metadata can influence the modeling process in terms of topic prevalence (to characterize documents) and topical content (to parameterize the distribution of word occurrences). We consider the covariates of the investor sentiment aggregated at investors' trading experience, holding strategy, asset category, bullish/bearish sentiment, and posted timestamp. In particular, investors' trading experience is employed as the covariate in topical content, while other covariates are engaged in topic prevalence.

A graphical illustration of the data generative process of structural topic modeling is provided in Figure 3. Given a topic *k* from the pre-fixed topic number *K*, we identify the relevant document *d* from all *D* documents (aggregated tweets). We denote the metadata as a matrix *X*, with each row (denoted *x*_*d*_) listing the values of metadata covariates for document *d*. The words of a corpus-level vocabulary list *V* are indexed by *v* ∈ {1, …, *V*}. The document *d* holds *N*_*d*_ empty positions, waiting to be filled by *N*_*d*_ words. To fulfill the word positions in a document, first, a specific topic-prevalence vector should be generated using the associated metadata (Grajzl and Murrell, 2019). Topic prevalence vector θ_*d*_ follows a Logistic Normal distribution with a mean vector parameterized as a function of covariates *x*_*d*_, shown in Equation (1):


(1) $$\begin{array}{l} \begin{array}{c} \theta_{d}\sim {\text{LogisticNormal}}_{k-1}\left(\Gamma^{\prime }x_{d}^{\prime },\Sigma \right), \end{array} \end{array}$$

where Γ′ is a matrix of coefficients and Σ is a general variance-covariance matrix. Given the topic-prevalence vector, we can draw one specific *z*_*d,n*_, which indicates the associated topic for the word *w*_*d,n*_ in the document, as shown in Equation (2):


(2) $$\begin{array}{l} \begin{array}{c} z_{d,n}\sim {\text{Multinomial}}_{K}\left(\theta_{d}\right), \end{array} \end{array}$$

Note that, when a specific topic *k* is chosen, the *k*-th entry of *z*_*d,n*_ is unity and all other entries are zero.

Topical content defines the distribution over terms associated with the topics as an exponential family model, added as a function of the marginal frequency of occurrence deviations for each term *m*_*v*_, and of deviations from different topics κ_*k,v*_, covariates κ_*y*_*d*_,*v*_ and their interactions κ_*y*_*d*_,*k,v*_ in Equation (3):


(3) $$\begin{array}{l} \begin{array}{c} \beta_{d,k,v}=\frac{exp\left(m_{v}+\kappa_{k,v}+\kappa_{y_{d},v}+\kappa_{y_{d},k,v}\right)}{\underset{v}{\sum }exp\left(m_{v}+\kappa_{k,v}+\kappa_{y_{d},v}+\kappa_{y_{d},k,v}\right)}, \end{array} \end{array}$$

where β_*d,k,v*_ is the probability of choosing vocabulary word *v* to fill a position in document *d* given topic *k*. Then a specific word *w*_*d,n*_ is chosen from the vocabulary list in Equation (4):


(4) $$\begin{array}{l} \begin{array}{c} w_{d,n}\sim {\text{Multinomial}}_{V}\left(Bz_{d,n}\right), \end{array} \end{array}$$

where *Bz*_*d,n*_ = [β_*d,k*,1_, …, β_*d,k,v*_, …, β_*d,k,V*_] is the overall probability of the words given topic *k*.

In short, the parameters mentioned above are estimated by solving the posterior likelihood maximization optimization problem via R's STM package (Roberts et al., 2019) which includes an iterative approximation-based variational expectation-maximization algorithm.

#### 4.2.2. The selection of topic numbers

**Figure 4:**
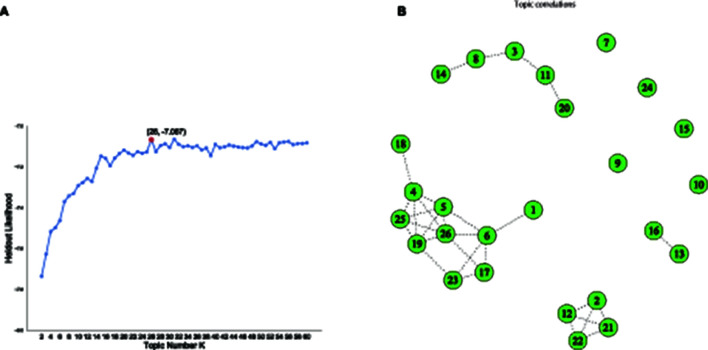
**(A)** Selecting optimal *k* topics by evaluating held-out likelihood with adjusting the number of topics from 2 to 60. **(B)** The correlation of 26 identified topics, exhibiting four connected clusters with relatively high marginal correlation, together with a few isolated topics.

In topic modeling, it is essential to determine a proper and the representative number of topics *K*, in order to uncover meaningful topics from the corpus. We perform an exhaustive search to identify the optimal *K* via the comparison of the held-out log-likelihood, which was used in the original paper of STM (Roberts et al., 2016) as a measure to understand the model fit, compare with other competing models and evaluate the comparative performance among these models. Essentially, the held-out likelihood indicates the probability of the held-out words appearing within a document. Those words are held out from the document in the estimation step and evaluated by the document-level latent variables after the model training (Wallach et al., 2009). More specifically, we estimate the STM model using the same configuration of covariates with varying *K* topic numbers, ranging from 2 to 60, to compute diagnostic properties of held-out log-likelihood, shown in Figure 4. As expected, higher numbers of the held-out log-likelihood indicate a more predictive model, and the held-out log-likelihood increases as the model become more complex with increasing topic numbers. In order to balance model complexity and topic quality, we choose to set topic number *K* as 26 because its estimated held-out log-likelihood seems to show better-organized topics than the ones with *K* = 25 or 27. In other words, adding one more topic in the modeling process does not yield more coherent topics, but the model's prediction performance instead worsens.

## 5. Results

### 5.1. Topic contents and correlations

By applying STM to the documents of aggregated tweets enriched with factors such as investor profiles and trading preferences, asset categories, and investor sentiment, as illustrated in Table 1, we identify 26 topics and summarize the semantic meaning of each topic on the grounds of the words mentioned in the highest marginal score words list, and meanwhile, appeared in the top rank of the highest marginal FREX words list. Following the metrics introduced in Roberts et al. (2019) and Chang (2015), the highest marginal score is obtained by dividing the log frequency of the word in the topic by the log frequency of the word in other topics as in Equation (5),


(5) $$\begin{array}{l} SCORE_{k,v}=\beta_{k,v}\left(\log\beta_{k,v}-\frac{1}{K}\underset{j}{\sum }\log\beta_{j,v}\right), \end{array}$$

while FREX (frequency and exclusivity) weights words by their overall frequency and how exclusive they are to the topic to determine the weighted harmonic mean as in Equation (6)


(6) $$\begin{array}{l} FREX_{k,v}={\left(\frac{w}{ECDF\left(\beta_{k,v}/\sum_{j=1}^{K}\beta_{j,v}\right)}+\frac{1-w}{ECDF\left(\beta_{k,v}\right)}\right)}^{-1}, \end{array}$$

where *w* is the weight for exclusivity (which we set to 0.5 as a default) and *ECDF* denotes the corresponding empirical CDF function.

**Table 1:** The semantic meanings (topic names) of 26 topics on the grounds of the words mentioned in the highest marginal score and FREX words list (keywords).

**Topic Name**	**Topic ID**	**Keywords**
Bearish Terms	Topic 1	put, bearish, dump, fall, short, pump, fail, fade, ugly, downside, lose, pose, tank, dead, lod
FDA Drug Approval (Ebola)	Topic 2	$amrn, $hlf, $ibio, $lak, $apt, $sgyp, $bgmd, $srpt, ebola, $rprx, $isrg, nce, fda, $avnr, $fold
Ukrainian Crisis (2014)	Topic 3	$ugaz, $uso, $qqq, $dgaz, $ibb,$tvix,$gdxj, $jnug, $xiv, etf, $rsx, $russ, $tan, $tna,$fxi
Green Tech	Topic 4	$plug, $bidu, $amd, $nok, $fslr,$gtat, $sina, plug, $cal, $fio, $jaso, $nihd, $renn, $yyg, $tsl
IT Companies	Topic 5	$aapl, $goog, $lnkd, $bidu, $sina, $znga, $ocz, $crm, $yelp, $msft,$clwr, $ibm, $grpn, $fio,$hpq
Online Service	Topic 6	$nflx, $amzn, $p, $cmg, $gevo, $gdp, $z, $rig, $chk, $wynn,$anf, $newl, $hero, $ymcd,$sdrl
Long Term Investment	Topic 7	dividend, growth, quarter, sale, beat, revenue, raise, guidance, investor, estimate, increase, inc, report, value, yield
ETF	Topic 8	$spi, $iwm, $uvxi, $qqq, $vxx, $dia, $tlt, $tza,$xlf, $tna, spi, vix, $faz,$fxi,$fa
Greece Crisis	Topic 9	$bac, $fnma, $nbg, $g, $jpm, $c, $aig, $drl, $wfc, bank, $fmcc,$m, $ocn, $kcg,$axp
Clean Energy	Topic 10	$hlf, $cln, $nflx,$fcel,$so,$exc, $optt, $nrg,$duk, ackman, util,$at, energy, $aep, herbalif
Precious Metal	Topic 11	$gld, $slv, $nugt,$gdx, $fcel, gold, $ung, silver, $gdxj,$agq, $dust,$htm, miner, etf, $uso
FDA Drug Approval (Vascepa)	Topic 12	$amrn, $arna, $qcor, $vvus, $clsn,$dndn, $hznp, fda, approve, $kerx, $srpt, $affi, $navb, $rosg,$sppi
Automobile	Topic 13	$tsla, $gmcr, $f, $lulu, $soda, $deck, $mnst, $lf, $pay,$nk, $ko,$dmnd, ford,$fosl,$wprt
Index	Topic 14	$spx, $dax, $djia, $vix, $rut, $compq, $usdx, $fts, $nya, $tnx, $dxi, $ndx, spx,$xli, index
Forex	Topic 15	forex, pip, euro, eurusd, usd, bearish, eur, pair, gbp, aud, fib, jpy, eunderf, ecb, elliottwave
Tesla & Electric Vehicle	Topic 16	$tsla, $kndi, $gmcr, $lulu, $app, $soda, tesla, $nk, $jakk, kndi,$ko, $sn, car, kandi, $nus
Retail Store	Topic 17	$jcp, $lv, $kor,$dang, $dri, $sbux, $v, $siri,$ma, $mgm,$exp,$imax,$cstr,$zagg,$yrcw
3-D Printing	Topic 18	$ddd, $bldp, $cat, $rgs, $hpj, $cpst, $ba, $xon,$idn, $jk, $artx, $rgr, $len,$hov, $cdti
Mobile Apps & Hardware	Topic 19	$twtr, $yhoo, $neon, $csiq, $mu, $yelp,$gtat, $amba, $dg,$baba,$himx,$jrjc,$swk, $otiv, $sun
Fossil Fuels	Topic 20	$wlt, $clf, $fcx, $hk, $aa, $xom, $peix, $pot, $anv, $bp, $cf, $lng, $usu $anr, $x,
Therapeutics	Topic 21	$aria, $ino, $rnn, $gild, $rxii, $chtp, $opk, $isr, $cytr, $icpt, $achn, $bioc, $gen, $bgmd, $idra
Bio-Pharmaceutical (diabetes)	Topic 22	$mnkd, $gild, $ino, $isr, $achn, $rxii, $cldx, $rmti, $cprx, $idra, $cytk, $pcyc, $icpt, $uni, $arwr
Travel	Topic 23	$gpro, $di, $l, $rsh, $fre, $rad, $jblu, $rada, $luv, gopro, $wfm, $fro, $esi, $cnet, $wyy
Bullish Terms	Topic 24	breakout, resist, week, flag, high, break, gap, setup, swing, min, entry, upside, vol, pattern, base
Mobile Internet	Topic 25	$aapl, $fb, $nok, $znga, $gluu, $amd, appl, iphon, $unxl, $invn, $bbri, $yhoo, $msft, $goog, $ttwo
Hotel Platform	Topic 26	$pcln, tmr, green, babi, load, offer, Monday, talk, novemb, huge, option, pick, need, worth, sign

**Figure 5:**
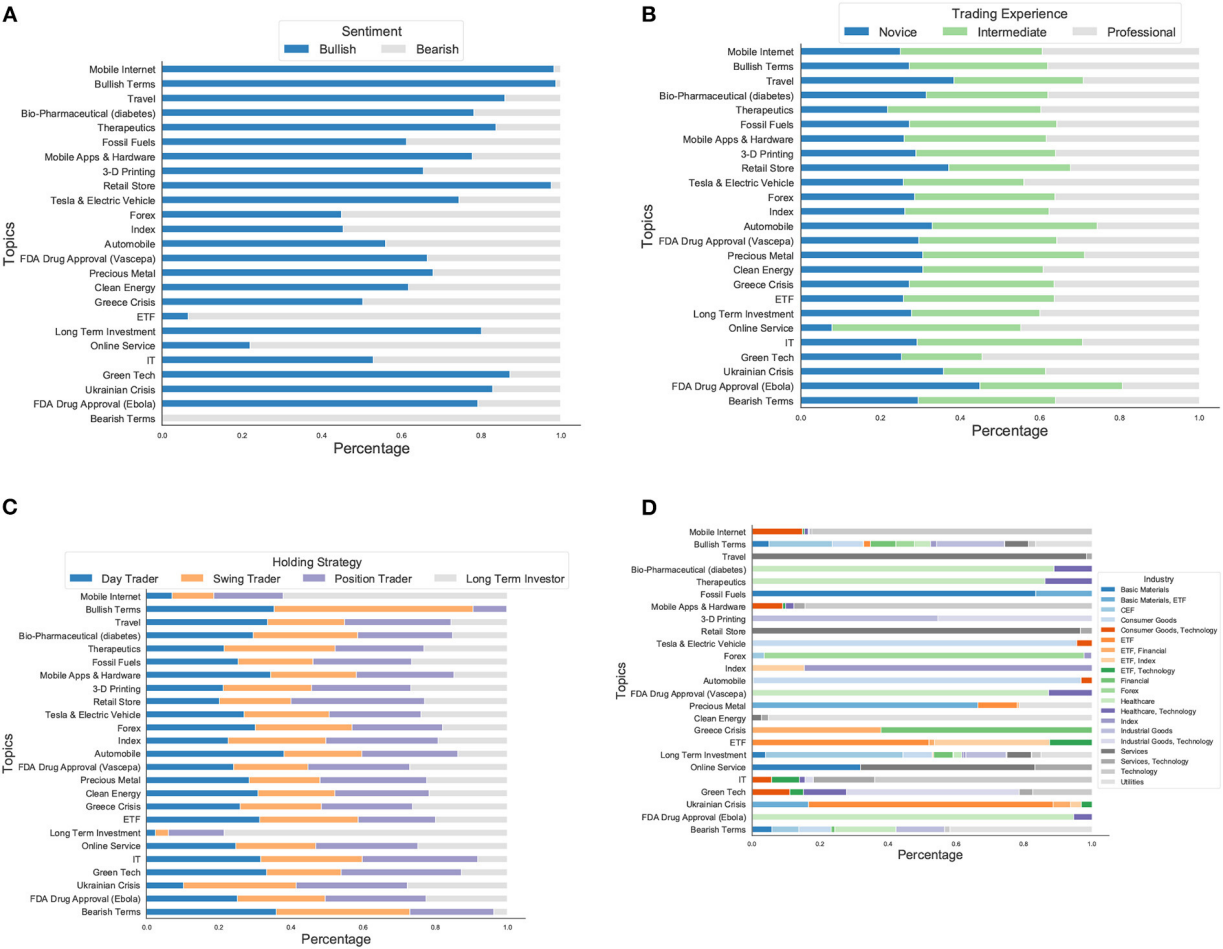
**(A)** The heterogeneity of investor sentiment in each topic in terms of bullish/bearish sentiment. The market sentiment is overall more bullish than bearish. **(B)** The heterogeneity of investor sentiment in each topic in terms of trading experience compositions. The investors with different trading experiences show considerable divergence on topics they are interested in. **(C)** The heterogeneity of investor sentiment in each topic in terms of holding strategy compositions. Investors with different trading strategies have divergent focuses and word preference across topics. **(D)** The distributions of asset categories across topics. Several topics are related to specific asset categories, and the belonging tweets contain discussions of the companies from the same asset category or associative industrial fields. Furthermore, we found that topics of important social events contain many company tickers that reveal the underlying associative asset categories under discussions of the event.

In conformance to the studies of event discovery, one primary approach for understanding the heterogeneity of investor sentiment is to separate the breaking finance news from the ongoing investment-related conversations (Stieglitz et al., 2018). Although the volume of data makes it challenging to discover the relevant news and events in dynamic social media communication (Kasiviswanathan et al., 2011), especially in a platform with words limitation, we found that the topics identified by STM have comprehensive coverage and compelling description of different news and asset categories. The topics not only reveal the discussions around specific asset categories in the financial market as expected, such as Forex, Index, and ETF, but also provide focus on shorter-term events and the resulting financial activities, such as FDA Drug Approval, Ukrainian Crisis (2014), 3-D Printing, and Greece Crisis. The correlation of the 26 identified topics, as revealed in Figure 5, exhibits four connected clusters with the relatively high marginal correlation of the mode of the variational distribution, together with a few isolated topics. More specifically, the largest cluster covers 10 topics about high-tech companies in different sectors. The second cluster contains five topics related to mining, natural resources, and financial derivatives. The third cluster relates to 4 bio-pharmaceutical and drug-related topics. The fourth cluster involves two topics about automobiles and electric cars. As such, the spread of investor sentiment across relevant topics may signal the flow of investor attention and discussions within topic clusters. Taking the second cluster as an example, we found that the high topic correlations between mining, natural resources, and financial derivatives were also observed and illustrated by Ji and Guo (2015). The study pointed to the strong influence of online market discussions on commodity prices (e.g., crude oil, heating oil, corn, and gold). It indicated that commodity markets could easily absorb the public concern of the information-sensitive traders, considering the fast transmission and frequent changes of market information.

### 5.2. Heterogeneity in investor sentiment

Moreover, the identified topics not only reveal the short-term social events and popular financial investment products of widespread concerns based on the text content but also unmask the heterogeneity of investor sentiment given document characteristics—the metadata (covariates) annotating the content. More specifically, we focused on the documents that contribute the most to each topic, i.e., whose topic proportions (θ) obtained after model estimation are at least larger than 50%, and then explored the distributions of the metadata of these documents on topic composition. The results in Figure 5 illustrate the heterogeneity of investor sentiment in each topic in terms of bullish/bearish sentiment (Figure 5A), compositions of investor experience (Figure 5B) and trading preference (Figure 5C), and the distributions of asset categories (Figure 5D).

The market sentiment shown in Figure 5A is more bullish than bearish overall. For example, except for technical terminologies, the sentiment is more bullish on topics about mobile internet, high tech companies, clean energy, and healthcare. Similarly, more investors hold a positive attitude toward the financial market for most topics associated with social events. Interestingly, investors who feel more optimistic about Ukrainian Crisis (2014) comprise more of the percentage than those for the Greek Crisis, possibly because the Greek Crisis has been a systemic financial crisis affecting many countries. At the same time, Ukrainian Crisis (2014) is a geopolitical problem around Ukraine, so it has less impact directly on the financial market. Furthermore, the bearish sentiment in ETF topics prevails over the bullish part, which is compatible with a study of the passive investment in ETF (Paudel et al., 2020), stating that the changes in negative sentiment have a more powerful effect on the ETF's downside risk in comparison to the changes in positive sentiment.

### 5.3. Heterogeneity in investor trading experience and trading strategy

Besides the divergence in investor sentiment, investors with various profiles tend to discuss financial markets with heterogeneous beliefs, resulting in different distributions regarding the topic prevalence and proportion. The investors with different trading experiences show considerable divergence on topics they are interested in, as shown in Figure 5B. Novice investors focus more on medicines for Ebola, travel, Retail Store, and Ukrainian Crisis (2014), and intermediate ones like to talk about Online Service and Automobile, while the professional comments more on the Green Tech, Tesla& Electric Vehicle, Online Service and use more market terminologies. In particular, a study of non-professional investors for using sustainability information in the investment decision-making (Hafenstein and Bassen, 2016) illustrates that non-professional investors are less sensitive to sustainable development and do not distinguish between the different aspects of sustainability. Moreover, when checking the highest weighted words under each level of trading experience, we found that the novice investors tend to mention more specific events in more detail, while the professional investors like to talk about the future tendencies of asset categories and the related financial instruments. For example, in the topic of the Greek Debt Crisis, novice investors like to use the words “greek,” “sentiment,” “JPMorgan,” “fanni” (Fannie Mae, the Federal National Mortgage Association), “freddi” (Freddie Mac, the Federal Home Loan Mortgage Corporation), “bac” (The Bank of America), “nbg” (National Bank of Greece), *etc*. For professional investors in the same topic, they mention more about “bank,” “finance,” “bac” (The Bank of America), “Genpact” (a global professional services firm that makes business transformation real), “American Express” (a multinational financial services corporation), “KCG" (an American global financial services firm engaging in market making, high-frequency trading, electronic execution, and institutional sales and trading), etc.

Moreover, investors with different trading strategies, analogous to those with different trading experiences, also have divergent focuses and word preferences across topics, as shown in **Figure 5C**. For example, day traders focus more on Forex, position traders and long term traders are most consistent across different topics and tend to more often use market terminologies related to long term investment (“dividend,” “quarter,” “growth,” “estim,” “raise,” “revenue,” “guidance,” “margin,” “forecast,” “undervalue,” *etc*.). By contrast, day traders and swing traders are more likely to discuss topics using sentiment-related words such as “breakout,” “posit,” “resist,” “high,” “bottom,” “ready,” “upside,” “bull” in the topic of bullish terms and “put,” “bearish,” “fall,” “short,” “dump,” “weak,” “downside” in the topic of bearish terms.

### 5.4. Heterogeneity in asset category

In terms of asset categories extracted from document contents, we found strong heterogeneity likewise, as shown in Figure 5D. First, several topics are related to specific asset categories, and the belonging tweets contain discussions of the companies from the same asset category or associative industrial fields. For example, on the topic of Therapeutics, medical and pharmaceutical companies such as Ariad Pharmaceuticals, Rexahn Pharmaceuticals, Inovio Pharmaceuticals and RXi Pharmaceuticals are highly mentioned by investors. Second, in some high-tech topics, such as mobile apps & hardware, 3-D printing, green tech, mobile internet and IT, several asset categories originally intersect in the industrial chains, composing from stem to stern. Therefore, companies from upstream and downstream asset categories are involved reasonably in one topic, further proving the validity of the model results. For instance, in the topic of 3-D printing, the key asset is 3D Systems, a company that manufactures 3-D printing materials and devices and offers 3-D printing services. Meanwhile, other assets along the supply chain of 3-D printing appear in the same topic, including energy fuel cell and storage, construction manufacturing, and retail processes, such as Ballard Power Systems (a global provider of innovative clean energy fuel cells), Caterpillar (an international construction-equipment manufacturer), Capstone Turbine (a gas turbine manufacturer that specializes in microturbine power), Highpower International (specialized in clean energy storage) and Intellicheck (a technology company which markets threat identification and identity authentication solutions for retail fraud prevention). As illustrated in Rehnberg and Ponte (2018), 3D printing could revolutionize production processes and restructure the global manufacturing value chain, which is captured by the topic results that cover the affected assets in different industrial sectors.

Furthermore, we found that topics of important social events contain many company tickers that reveal the underlying associative asset categories under discussions of the event. For example, in the Ukrainian Crisis (2014) topic, investors mention assets of basic materials and customer goods heavily. One possible explanation is that the sanctions against Russia severely impacted these assets after the Ukrainian Crisis (2014) (Nivorozhkina and Castagneto-Gissey, 2016), which we may repeatedly observe in conversations related to more recent Ukrainian geopolitical events. In another example of the Greek Crisis, finance and ETFs are the categories of main discussions. According to a study of ETFs and Index Funds in the Greek Market before and during the Crisis (Rompotis, 2013), the funds experienced significant losses over the economic crisis period, when the International equities market was very turbulent. As such, our model results may indicate the heterogeneity of social event topics in terms of the asset categories that may be affected.

### 5.5. Relationship between metadata and topics

**Figure 6:**
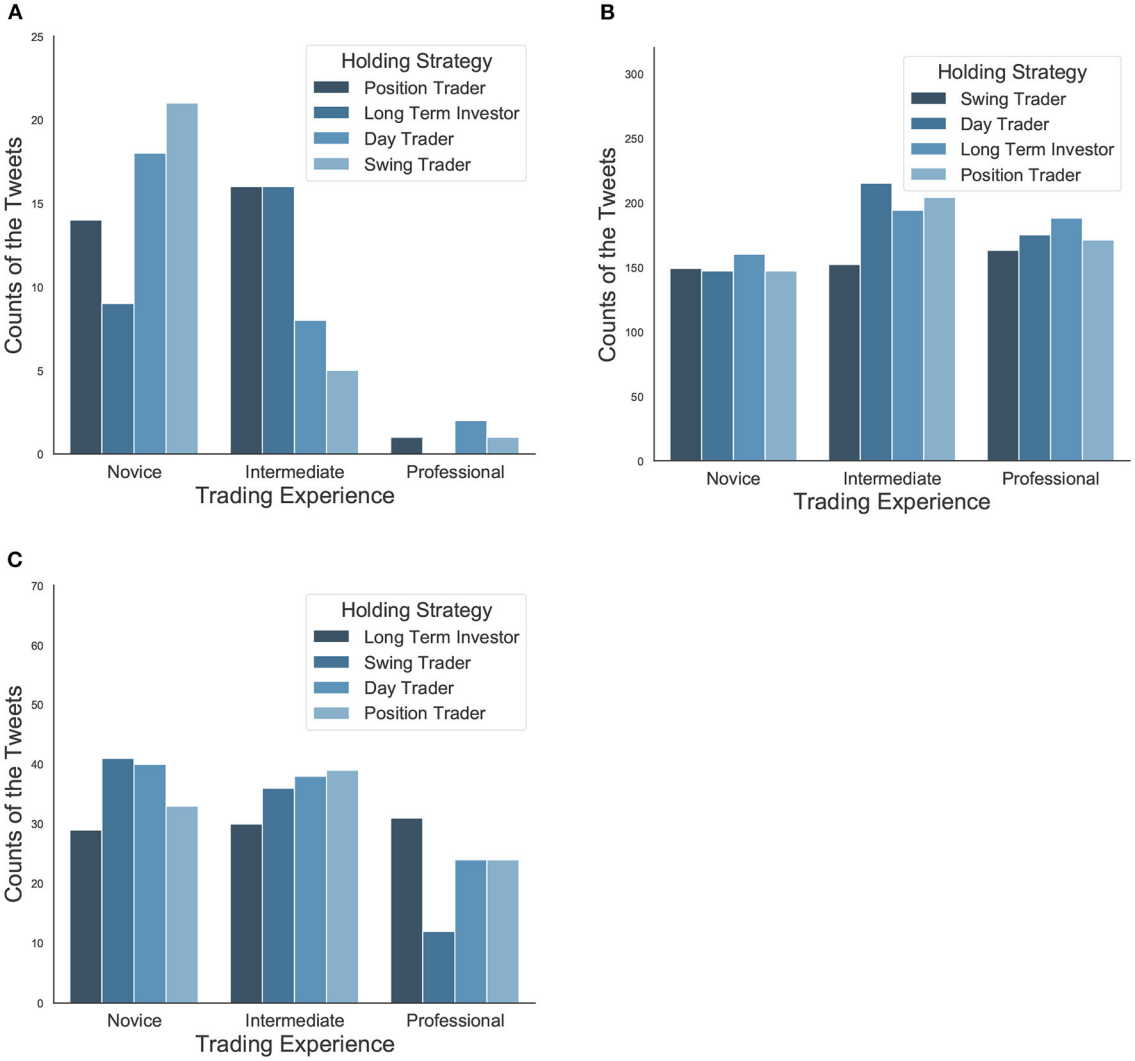
**(A)** The distribution of tweet proportion according to investor trading experience and holding strategy on Topic 2 [FDA Drug Approval (Vascepa)]. Novice investors are more likely to discuss this topic than intermediate and professional traders. **(B)** The distribution of tweet proportion according to investor trading experience and holding strategy on Topic 9 (Greek Debt Crisis). It is a hot topic for all types of investors with different profiles in terms of a large number of tweets. **(C)** The distribution of tweet proportion according to investor trading experience and holding strategy on Topic 16 (Tesla & Electric Vehicles). Within the professional investors, those who follow long-term strategy post a significant amount than the swing traders in Tesla and the electric vehicle area, which proves they indeed have a good more developed sense of selecting stocks under consideration of the current stock prices of Tesla and the potential electric vehicles.

In order to further examine the relationship between metadata and topics, we estimated a generalized linear model with documents as the observations. The dependent variable is the proportion of each document about a specific topic derived from the STM model and the covariates are document-level metadata. For the sake of space, we selected three exemplary topics (FDA Drug Approval (Vascepa), Greek Debt Crisis and Tesla & Electric Vehicles), and show the estimated results in Table 2. We also illustrate the distribution of tweet proportion according to investor trading experience and holding strategy in Figure 6.

**Table 2:** The generalized linear model regression estimation results with statistical significance of three exemplary topics (FDA Drug Approval (Vascepa), Greek Debt Crisis and Tesla & Electric Vehicles) based on document-level covariates under study.

	**(a)**	**(b)**	**(c)**
	**FDA drug approval (vascepa)**	**Greek debt crisis**	**Tesla & Electric vehicles**
TradingExperience: Novice	0.0050^***^	0.0019	–0.0007
	(0.0013)	(0.0012)	(0.0016)
TradingExperience: Professional	0.0006	–0.0030^**^	0.0052^***^
	(0.0013)	(0.0011)	(0.0016)
HoldingStrategy: Long Term Investor	0.0036^*^	0.0031^*^	–0.0003
	(0.0014)	(0.0014)	(0.0016)
HoldingStrategy: Position Trader	0.0031^*^	–0.0027^*^	0.0010
	(0.0014)	(0.0013)	(0.0017)
HoldingStrategy: Swing Trader	0.0007	–0.0061^***^	0.0009
	(0.0013)	(0.0014)	(0.0015)
AssetCategory: Basic Materials	–0.0024	–0.0006	0.0017
	(0.0028)	(0.0023)	(0.0031)
AssetCategory: CEF	0.0016	0.0025	–0.0007
	(0.0033)	(0.0027)	(0.0036)
AssetCategory: Consumer Goods, Technology	0.0006	–0.0002	0.2022^***^
	(0.0030)	(0.0025)	(0.0061)
AssetCategory: Consumer Goods	–0.0024	–0.0006	0.2985^***^
	(0.0028)	(0.0023)	(0.0056)
AssetCategory: ETF, Financial	0.0006	0.5264^***^	0.0002
	(0.0030)	(0.0056)	(0.0033)
AssetCategory: ETF, Index	–0.00002	0.0002	0.0003
	(0.0029)	(0.0025)	(0.0032)
AssetCategory: ETF, Technology	0.0002	0.0002	0.0002
	(0.0029)	(0.0025)	(0.0033)
AssetCategory: ETF	–0.0024	–0.0006	0.0017
	(0.0028)	(0.0023)	(0.0031)
AssetCategory: Financial	–0.0025	0.5736^***^	0.0018
	(0.0028)	(0.0039)	(0.0031)
AssetCategory: Forex	–0.0016	–0.00001	0.0016
	(0.0029)	(0.0024)	(0.0032)
AssetCategory: Healthcare, Technology	0.1580^***^	–0.000003	–0.0012
	(0.0045)	(0.0026)	(0.0034)
AssetCategory: Healthcare	0.2805^***^	–0.0006	0.0016
	(0.0050)	(0.0023)	(0.0031)
AssetCategory: Index	–0.0011	–0.0001	0.0011
	(0.0029)	(0.0024)	(0.0032)
AssetCategory: Industrial Goods, Technology	0.0017	–0.0003	–0.0016
	(0.0031)	(0.0026)	(0.0034)
AssetCategory: Industrial Goods	–0.0021	–0.0005	0.0015
	(0.0028)	(0.0024)	(0.0031)
AssetCategory: Services, Technology	–0.0016	–0.0002	0.0015
	(0.0028)	(0.0024)	(0.0031)
AssetCategory: Services	–0.0025	–0.0006	0.0018
	(0.0028)	(0.0023)	(0.0031)
AssetCategory: Technology	–0.0026	–0.0007	0.0019
	(0.0028)	(0.0024)	(0.0031)
AssetCategory: Utilities	0.000007	–0.0001	–0.0002
	(0.0029)	(0.0025)	(0.0033)
Sentiment: Bullish	0.0043^***^	0.0028^**^	0.0055^***^
	(0.0010)	(0.0009)	(0.0013)
(Intercept)	0.0052	0.0027	–0.0080
	(0.0055)	(0.0046)	(0.0059)

* p < 0.05,

** p < 0.01, and

*** p < 0.001.

According to the regression results shown in Table 2a, as expected, the first topic of FDA Drug Approval (Vascepa) is positively related to assets from the healthcare sector as well as from both the healthcare and technology sector. Interestingly, novice investors are more likely to discuss this topic than intermediate and professional traders. This finding is consistent with a study of forty firms from the chemical and drug industries (Barron et al., 2004), which suggested that non-professional investors are encouraged by SEC's “Fair Disclosure" regulation and thus can generate more diverse private information and increase precision in their individual forecasts. However, such disclosure had no significant effect on professional investors, because they could extract the information from low and high disclosure regimes. In particular, Figure 6A shows that day traders and swing traders are over-represented among novice investors, implying that news about drug approval draws more attention from short-term traders.

The second topic of the Greek Debt Crisis is a hot topic for all types of investors with different profiles in terms of a large number of tweets (Figure 6B). Based on the results shown in Table 2b, this topic is positively associated with assets in the finance and ETF category. Professional investors and position and swing traders tend to discuss this topic less, while long-term traders are more likely to contribute to it.

For the third topic on Tesla & Electric Vehicles, Table 2c shows that it is related to consumer goods and technology, which agrees with the main products of the company Tesla. Professional traders focus on this topic more heavily, and the market, in general, is significantly bullish. Also, among the professional investors, those who follow long-term strategy post a significant amount than the swing traders in Tesla & Electric vehicle area, which proves they indeed have a good more developed sense of selecting stocks under consideration of the current stock prices of Tesla and the potential electric vehicles (Figure 6C). This finding supports the statement in a study related to behavior contagion among investors (Fallahgoul and Lin, 2020), that fundamental, professional, and swing traders are the most influential investors and leading in information cascades within investment approach, experience, and holding period categories, respectively. In contrast, momentum, novice, and position traders account for the least.

## 6. Conclusions

Although investor sentiment may have a significant heterogeneous effect on the financial market, the sentiment analysis in the finance industry typically produces aggregated investor sentiment index without accounting for the heterogeneity. For example, in the WallStreetBets vs. Wall Street saga, investor sentiment is highly driven by heterogeneous investor profiles, as the topic is disproportionately contributed by bullish sentiment from non-professional retail investors that mainly engage in day trading (Umar et al., 2021)–as such, extracting investor sentiment around this topic should reflect the underlying heterogeneity.

This paper provides empirical evidence on the heterogeneity of investor sentiment on social media. More specifically, we examine the interactions between investor sentiment and multiple finance-specific factors and identify the thematic content of investor sentiment across investor profiles, bullish/bearish sentiment, and categories of mentioned assets. From identified topical content from a large-scale social media dataset dedicated to investors sharing their opinions, we find these topics have different compositions in terms of trading experience, trading strategy, sentiment, and asset category. This implies that investors' trading preferences may lead them to allocate their attention to different assets with heterogeneous sentiments. Thus, we believe that investor sentiment should be used by considering factors such as investor profiles instead of simple aggregation.

This paper is limited to discussing the content heterogeneity of investor sentiment and does not drill down into the mechanism that how different types of investors may shape the online discussion on social media and its impact on the financial market. For future work, we will study how to use the identified topics to perform more efficient sentiment analysis, e.g., which investor profiles may produce more meaningful sentiment for the specific event and which industry sectors it may affect. Then, we will explore efficient investor sentiment extraction and aggregation that may account for the heterogeneity of investor sentiment across different dimensions that may have different impacts on the financial market. Last, we will examine the relationship between the factors that drive heterogeneous investor sentiment and topic extraction, in order to validate the theoretical foundation in finance research.

## Data availability statement

The original contributions presented in the study are included in the article/supplementary materials, further inquiries can be directed to the corresponding author.

## Author contributions

RJ performed data analysis, build the model, and prepared the manuscript. QH performed data analysis, build the model, and prepared the manuscript. Both authors contributed to the article and approved the submitted version.

## Funding

This work was funded by Fundação para a Ciência e a Tecnologia (UID/ECO/00124/2019, UIDB/00124/2020 and Social Sciences Data Lab, PINFRA/22209/2016), POR Lisboa and POR Norte (Social Sciences Data Lab, PINFRA/22209/2016), and European Union's Horizon 2020 Research and Innovation Programme under the Marie Skłodowska Curie grant agreement No 812912 for the project BIGMATH.

## Conflict of interest

The authors declare that the research was conducted in the absence of any commercial or financial relationships that could be construed as a potential conflict of interest.

## Publisher's note

All claims expressed in this article are solely those of the authors and do not necessarily represent those of their affiliated organizations, or those of the publisher, the editors and the reviewers. Any product that may be evaluated in this article, or claim that may be made by its manufacturer, is not guaranteed or endorsed by the publisher.

## Footnotes

^1^StockTwits maintains a list of nearly 8,000 ticker symbols and corresponding categories that is used for monitoring the conversations around the assets. Stocks are further categorized as Financial, Technology, Services, Basic Materials, Healthcare, Consumer Goods, Industrial Goods, Utilities.

## References

[B1] Antweiler W. Frank Z. M. (2004). Is all that talk just noise? The information content of internet stock message boards. J. Finan. 59, 1259–1294. 10.1111/j.1540-6261.2004.00662.x

[B2] Audrino F. Sigrist F. Ballinari D. (2020). The impact of sentiment and attention measures on stock market volatility. Int. J. Forecast. 36, 334–357. 10.1016/j.ijforecast.2019.05.010

[B3] Baik B. Cao Q. Choi S. Kim J.-M. (2016). Local Twitter Activity and Stock Returns. SSRN Working Paper, No: 2783670.

[B4] Baker M. Wurgler J. (2007). Investor sentiment in the stock market. J. Econ. Perspect. 21, 129–151. 10.1257/jep.21.2.129

[B5] Bar-Haim R. Dinur E. Feldman R. Fresko M. Goldstein G. (2011). Identifying and following expert investors in stock microblogs, in Proceedings of the Conference on Empirical Methods in Natural Language Processing (Seattle, WA), 1310–1319.

[B6] Barron O. E. Byard D. Enis C. R. (2004). Leveling the informational playing field. Rev. Account. Finan. 3, 21–46. 10.1108/eb04341217500506

[B7] Bartov E. Faurel L. Mohanram P. S. (2018). Can twitter help predict firm-level earnings and stock returns? Account. Rev. 93, 51865. 10.2308/accr-51865

[B8] Besbris M. Schachter A. Kuk J. (2021). The unequal availability of rental housing information across neighborhoods. Demography 58, 1197–1221. 10.1215/00703370-9357518 34196705

[B9] Blei D. Ng A. Jordan M. (2003). Latent dirichlet allocation. J. Mach. Learn. Res. 3, 993–1022. 10.1162/jmlr.2003.3.4-5.993

[B10] Blei D. M. Lafferty J. D. (2006). Dynamic topic models, in Proceedings of the 23rd International Conference on Machine Learning, ICML '06 (New York, NY: Association for Computing Machinery), 113–120.

[B11] Bollen J. Mao H. Zeng X. (2011). Twitter mood predicts the stock market. J. Comput. Sci. 2, 1–8. 10.1016/j.jocs.2010.12.007

[B12] Boudoukh J. Feldman R. Kogan S. Richardson M. (2013). Which News Moves Stock Prices? A Textual Analysis. NBER Working Paper, No. 18725.

[B13] Brown N. C. Crowley R. M. Elliott W. B. (2020). What are you saying? Using topic to detect financial misreporting. J. Account. Res. 58, 237–291. 10.1111/1475-679X.12294

[B14] Cerchiello P. Nicola G. (2018). Assessing news contagion in finance. Econometrics 6, 1–19. 10.3390/econometrics6010005

[B15] Chang J. (2015). lda: Collapsed Gibbs Sampling Methods for Topic Models. R Package Version 1.4.

[B16] Chen H. De P. Hu Y. J. Hwang B.-H. (2014). Wisdom of crowds: the value of stock opinions transmitted through social media. Rev. Finan. Stud. 27, 1367–1403. 10.1093/rfs/hhu001

[B17] Cookson J. A. Niessner M. (2020). Why don't we agree? Evidence from a social network of investors. J. Finan. 75, 173–228. 10.1111/jofi.12852

[B18] Curme C. Zhuo Y. D. Moat H. S. Preis T. (2017). Quantifying the diversity of news around stock market moves. J. Netw. Theory Finan. 3, 1–20. 10.21314/JNTF.2017.027

[B19] Curry T. A. Fix M. P. (2019). May it please the twitterverse: The use of twitter by state high court judges. J. Inf. Technol. Polit. 16, 379–393. 10.1080/19331681.2019.1657048

[B20] Dandapani K. Sabherwal S. Sarkar S. K. Zhang Y. (2008). Online talk: does it matter? Manag. Finan. 34, 423–436. 10.1108/03074350810872813

[B21] Das S. R. Chen M. Y. (2007). Yahoo! for Amazon: sentiment extraction from small talk on the web. Manag. Sci. 53, 1375–1388. 10.1287/mnsc.1070.0704

[B22] DeLong J. B. Shleifer A. Summers L. H. Waldmann R. J. (1990). Noise trader risk in financial markets. J. Polit. Econo. 96, 703–738. 10.1086/261703

[B23] Deng S. Huang Z. J. Sinha A. P. Zhao H. (2018). The interaction between microblog sentiment and stock returns: an empirical examination. MIS Q. 42, 895–918. 10.25300/MISQ/2018/14268

[B24] Doyle G. Elkan C. (2009). Financial topic models, in NIPS 2009 Workshop on Applications of Topic Models: Text and Beyond (Whistler, BC), 1–4.

[B25] Dyer T. Lang M. Stice-Lawrence L. (2017). The evolution of 10-K textual disclosure: evidence from latent dirichlet allocation. J. Account. Econ., 64, 2-). 10.1016/j.jacceco.2017.07.002

[B26] Fallahgoul H. Lin X. (2020). Who influences whom? behavior contagion among investors. SSRN Working Paper, No. 3764238.

[B27] Gan B. Alexeeva V. Bird R. Yeung D. (2020). Sensitivity to sentiment: news vs social media. Int. Rev. Finan. Anal. 67, 101390. 10.1016/j.irfa.2019.101390

[B28] Giannini R. Irvine P. Shu T. (2018). Nonlocal disadvantage: an examination of social media sentiment. Rev. Asset Pricing Stud. 8, 293–336. 10.1093/rapstu/rax020

[B29] Giannini R. C. Irvine P. J. Shu T. (2019). The convergence and divergence of investors' opinions around earnings news: evidence from a social network. J. Finan. Markets 42, 94–120. 10.1016/j.finmar.2018.12.003

[B30] Grajzl P. Murrell P. (2019). Toward understanding 17th century english culture: a structural topic model of francis bacon's ideas. J. Comp. Econ. 47, 111–135. 10.1016/j.jce.2018.10.004

[B31] Hafenstein A. Bassen A. (2016). Influences for using sustainability information in the investment decision-making of non-professional investors. J. Sustain. Finan. Invest. 6, 186–210. 10.1080/20430795.2016.1203598

[B32] Heston S. L. Sinha N. R. (2017). News versus sentiment: predicting stock returns from news stories. Finan. Anal. J. 73, 67–83. 10.2469/faj.v73.n3.3

[B33] Hill S. Ready-Campbell N. (2011). Expert stock picker: the wisdom of (Experts in) crowds. Int. J. Electron. Commerce 15, 73–102. 10.2753/JEC1086-4415150304

[B34] Hisano R. Sornette D. Mizuno T. Ohnishi T. Watanabe T. (2013). High quality topic extraction from business news explains abnormal financial market volatility. PLoS ONE 8, e64846. 10.1371/journal.pone.0064846 23762258 PMC3675119

[B35] Hong L. Davison B. D. (2010). Empirical study of topic modeling in Twitter, in Proceedings of the First Workshop on Social Media Analytics, SOMA'10 (Washington, DC), 80–88. 10.1145/1964858.1964870

[B36] Hou T. Tripathi A. (2015). The effect of social media on market liquidity, in Proceedings of Thirty-Fifth International Conference on Information Systems (Texas: ICIS), 1–10.

[B37] Huang A. H. Lehavy R. Zang A. Y. (2018). Analyst information discovery and interpretation roles: a topic modeling approach. Manag. Sci. 64, 2833–2855. 10.1287/mnsc.2017.2751

[B38] Ji Q. Guo J.-F. (2015). Market interdependence among commodity prices based on information transmission on the internet. Physica A 426, 35–44. 10.1016/j.physa.2015.01.054

[B39] Kasiviswanathan S. P. Melville P. Banerjee A. Sindhwani V. (2011). Emerging topic detection using dictionary learning, in 20th ACM International Conference on Information and Knowledge Management (Glasgow: ACM), 745–754.

[B40] Kim S. H. Kim D. (2014). Investor sentiment from internet message postings and the predictability of stock returns. J. Econ. Behav. Organ. 107, 708–729. 10.1016/j.jebo.2014.04.015

[B41] Lachana I. Schröder D. (2022). Investor sentiment, social media and stock returns: wisdom of crowds or power of words? SSRN Working Paper, No. 3842039.

[B42] Li Q. Wang T. Li P. Liu L. Gong Q. Chen Y. (2014). The effect of news and public mood on stock movements. Inf. Sci. 278, 826–840. 10.1016/j.ins.2014.03.096

[B43] Li W. Yin J. Chen H. (2017). Supervised topic modeling using hierarchical dirichlet process-based inverse regression: experiments on e-commerce applications. IEEE Trans. Knowl. Data Eng. 30, 1192–1205. 10.1109/TKDE.2017.2786727

[B44] Li X. Hendler J. A. Teall J. L. (2016). Investor attention on the social web. J. Behav. Finan. 17, 45–59. 10.1080/15427560.2015.1095752

[B45] Long C. Lucey B. M. Yarovaya L. (2021). 'I Just Like the Stock' versus 'Fear and Loathing on Main Street': The Role of Reddit Sentiment in the GameStop Short Squeeze. SSRN Working Paper, No: 3822315.

[B46] Loughran T. McDonald B. (2011). When is a liability not a liability? Textual analysis, dictionaries, and 10-Ks. J. Finan. 65, 35–65. 10.1111/j.1540-6261.2010.01625.x

[B47] Loughran T. Mcdonald B. (2016). Textual analysis in accounting and finance: a survey. J. Account. Res. 54, 1187–1230. 10.1111/1475-679X.1212334574884

[B48] Mai D. Pukthuanthong K. (2021). Economic narratives and market outcomes: a semi-supervised topic modeling approach. SSRN Working Paper, No. 3990324.

[B49] Mcauliffe J. Blei D. (2007). Supervised topic models, in Advances in Neural Information Processing Systems, Vol. 20, eds Platt J. Koller D. Singer Y. Roweis S. (Vancouver, BC: Curran Associates, Inc.).

[B50] Mehrotra R. Sanner S. Buntine W. Xie L. (2013). Improving LDA topic models for microblogs via tweet pooling and automatic labeling, in Proceedings of the 36th International ACM SIGIR Conference on Research and Development in Information Retrieval, SIGIR' 13 (Dublin), 889–892.

[B51] Nasseri A. A. Tucker A. De Cesare S. (2015). Quantifying StockTwits semantic terms' trading behavior in financial markets: an effective application of decision tree algorithms. Expert Syst. Appl., 42, 9192–9210. 10.1016/j.eswa.2015.08.008

[B52] Nguyen T. H. Shirai K. (2015). Topic modeling based sentiment analysis on social media for stock market prediction, in Proceedings of the 53rd Annual Meeting of the Association for Computational Linguistics (Beijing), 1354–1364.

[B53] Nivorozhkina E. Castagneto-Gissey G. (2016). Russian stock market in the aftermath of the Ukrainian crisis. Russ. J. Econ. 2, 23–40. 10.1016/j.ruje.2016.04.002

[B54] Nofer M. Hinz O. (2014). Are crowds on the internet wiser than experts? The case of a stock prediction community. J. Bus. Econ. 84, 303–338. 10.1007/s11573-014-0720-x

[B55] Oh C. Sheng O. R. L. (2011). Investigating predictive power of stock micro blog sentiment in forecasting future stock price directional movement, in Proceedings of the International Conference on Information Systems (ICIS) (Shanghai: ICIS), 1–19.

[B56] Oliveira N. Cortez P. Areal N. (2013). On the predictability of stock market behavior using stocktwits sentiment and posting volume, in Progress in Artificial Intelligence (Berlin; Heidelberg: Springer), 355–365.

[B57] Paudel K. Hassan M. K. Naka A. (2020). Consumer sentiment, demographics, and the downside risk: evidence from the passive investment in etfs. SSRN Working Paper, No. 3522200.

[B58] Pi neiro-Chousa J. R. López-Cabarcos M. Á. Pérez-Pico A. M. (2016). Examining the influence of stock market variables on microblogging sentiment. J. Bus. Res. 69, 2087–2092. 10.1016/j.jbusres.2015.12.013

[B59] Rabinovich M. Blei D. (2014). The inverse regression topic model, in International Conference on Machine Learning (Beijing: PMLR), 199–207.

[B60] Ramage D. Hall D. Nallapati R. Manning C. D. (2009). Labeled lda: a supervised topic model for credit attribution in multi-labeled corpora, in Proceedings of the 2009 Conference on Empirical Methods in Natural Language Processing (Singapore), 248–256.

[B61] Ranco G. Aleksovski D. Caldarelli G. Grcar M. Mozeti I. (2015). The effects of twitter sentiment on stock price returns. PLoS ONE 10, e0138441. 10.1371/journal.pone.0138441 26390434 PMC4577113

[B62] Rehnberg M. Ponte S. (2018). From smiling to smirking? 3d printing, upgrading and the restructuring of global value chains. Glob. Networks 18, 57–80. 10.1111/glob.12166

[B63] Ren J. Dong H. Padmanabhan B. Nickerson J. V. (2021). How does social media sentiment impact mass media sentiment? a study of news in the financial markets. J. Assoc. Inf. Sci. Technol. 71, 183–1197. 10.1002/asi.24477

[B64] Roberts M. E. Stewart B. M. Airoldi E. M. (2016). A model of text for experimentation in the social sciences. J. Am. Stat. Assoc. 111, 988–1003. 10.1080/01621459.2016.1141684

[B65] Roberts M. E. Stewart B. M. Nielsen R. A. (2020). Adjusting for confounding with text matching. Am. J. Pol. Sci. 64, 887–903. 10.1111/ajps.12526

[B66] Roberts M. E. Stewart B. M. Tingley D. (2019). Stm: an R package for structural topic models. J. Stat. Softw. 91, 1–40. 10.18637/jss.v091.i02

[B67] Rodrigues F. Lourenco M. Ribeiro B. Pereira F. C. (2017). Learning supervised topic models for classification and regression from crowds. IEEE Trans. Pattern Anal. Mach. Intell. 39, 2409–2422. 10.1109/TPAMI.2017.2648786 28103190

[B68] Rodriguez M. Y. Storer H. (2020). A computational social science perspective on qualitative data exploration: using topic models for the descriptive analysis of social media data. J. Technol. Hum. Serv. 38, 54–86. 10.1080/15228835.2019.1616350

[B69] Rompotis G. G. (2013). ETFs vs. Index funds in the greek market before and during the crisis. J. Index Invest. 4, 42–49. 10.3905/jii.2013.4.3.042

[B70] Shirokanova A. Silyutina O. (2018). Internet regulation media coverage in russia: topics and countries, in Proceedings of the 10th ACM Conference on Web Science, WebSci '18 (Amsterdam: ACM), 359–363.

[B71] Siehndel P. Gadiraju U. (2016). Unlock the stock: user topic modeling for stock market analysis, in CEUR Workshop Proceedings, Vol. 1558 (Bordeaux), 0–6.

[B72] Sprenger T. O. Tumasjan A. Sandner P. G. Welpe I. M. (2014). Tweets and trades : the information content of stock microblogs. Eur. Finan. Manag. 20, 926–957. 10.1111/j.1468-036X.2013.12007.x

[B73] Stieglitz S. Mirbabaie M. Ross B. Neuberger C. (2018). Social media analytics-challenges in topic discovery, data collection, and data preparation. Int. J. Inf. Mwanag. 39, 156–168. 10.1016/j.ijinfomgt.2017.12.002

[B74] Taddy M. (2013). Multinomial inverse regression for text analysis. J. Am. Stat. Assoc. 108, 755–770. 10.1080/01621459.2012.73416828694118

[B75] Tan S. D. Tas O. (2021). Social media sentiment in international stock returns and trading activity. J. Behav. Finan. 22, 221–234. 10.1080/15427560.2020.1772261

[B76] Tetlock P. C. (2007). Giving content to investor sentiment : the role of media in the stock market. J. Finan. 62, 1139–1168. 10.1111/j.1540-6261.2007.01232.x

[B77] Tetlock P. C. Saar-tsechansky M. Macskassy S. (2008). More than words: quantifying language to measure firms' fundamentals. J. Finan. 63, 1437–1467. 10.1111/j.1540-6261.2008.01362.x

[B78] Tu W. Cheung D. W. Mamoulis N. Yang M. Lu Z. (2016). Investment recommendation using investor opinions in social media, in Proceedings of the 39th International ACM SIGIR Conference on Research and Development in Information Retrieval-SIGIR '16, 881–884.

[B79] Tumarkin R. Whitelaw R. F. (2001). News or noise? Internet message board activity and stock prices. Finan. Anal. J. 57, 41–51. 10.2469/faj.v57.n3.2449

[B80] U.S. Securities Exchange Commission. (2013). Securities and Exchange Commission. SEC Says Social Media OK For Company Announcements If Investors are Alerted. Washington, DC: U.S. Securities Exchange Commission. Available online at: https://www.sec.gov/news/press-release/2013-2013-51htm

[B81] Umar Z. Gubareva M. Yousaf I. Ali S. (2021). A tale of company fundamentals vs sentiment driven pricing: the case of gamestop author links open overlay panel. J. Behav. Exp. Finan. 30, 100501. 10.1016/j.jbef.2021.100501

[B82] Wallach H. M. Murray I. Salakhutdinov R. Mimno D. (2009). Evaluation methods for topic models, in Proceedings of the 26th Annual International Conference on Machine Learning, ICML '09 (Montreal, QC), 1105–1112.

[B83] Wang C. Blei D. Heckerman D. (2012). Continuous time dynamic topic models. arXiv preprint arXiv:1206.3298. 10.48550/arXiv.1206.3298

[B84] Wang G. Wang T. Wang B. Sambasivan D. Zhang Z. (2015). Crowds on wall street : extracting value from collaborative investing platforms, in Proceedings of the 18th ACM Conference on Computer Supported Cooperative Work &Social Computing-CSCW'15 (Vancouver, BC: ACM), 17–30.

[B85] Xu S. X. Zhang X. (2013). Impact of wikipedia on market information environment: evidence on management disclosure and investor reaction. MIS Q. 37, 1043–1068. 10.25300/MISQ/2013/37.4.03

[B86] Zhang Y. Swanson P. E. Prombutr W. (2012). Measuring effects on stock returns of sentiment indexes created from stock message boards. J. Finan. Res. 35, 79–114. 10.1111/j.1475-6803.2011.01310.x

[B87] Zheludev I. Smith R. Aste T. (2014). When can social media lead financial markets? Sci. Rep. 4, 4213. 10.1038/srep04213 24572909 PMC5379406

[B88] Zhu J. Ahmed A. Xing E. P. (2012). MedLDA: maximum margin supervised topic models. J. Mach. Learn. Res. 13, 2237–2278. 10.5555/2503308.2503315

